# Expression of integrin-binding protein Nischarin in metastatic breast cancer

**DOI:** 10.3892/mmr.2015.3373

**Published:** 2015-02-18

**Authors:** JIE CHEN, WEI-LIANG FENG, WEN-JU MO, XIAO-WEN DING, SHANG-NAO XIE

**Affiliations:** Department of Breast Surgery, Zhejiang Cancer Hospital, Hangzhou, Zhejiang 310022, P.R. China

**Keywords:** Nischarin, breast cancer, metastasis

## Abstract

The present study aimed to investigate the expression of Nischarin protein in primary breast cancer (PBC), and to evaluate its role in tumor metastasis. Paired specimens of breast cancer tissues and adjacent normal tissues were surgically obtained from 60 patients with PBC at the Zhejiang Cancer Hospital (Hangzhou, China). Nischarin protein concentrations were determined by an ELISA assay. Breast cancer tissues exhibited a significantly lower concentration of Nischarin (5.86±3.19 ng/ml) compared with that of the adjacent noncancerous tissues (9.25±3.65 ng/ml; P<0.001). Furthermore, cancer tissue from patients with lymph node metastasis had significantly lower levels of Nischarin protein (4.69±2.40 ng/ml) than those of patients without lymph node metastasis (7.04±3.47 ng/ml; P=0.004). There was no significant difference in Nischarin protein expression levels between patients with grade I, II or III PBC (grade I, 5.44±3.57 ng/ml; grade II, 6.42±3.85 ng/ml and grade III, 5.10±1.18 ng/ml; P=0.765). The significant differences in the expression of Nischarin between: i) Cancer tissue and noncancerous tissue and ii) patients with and without lymph node metastasis, suggested that Nischarin may have a significant role in tumor occurrence and metastasis of breast cancer. Nischarin expression may therefore be used as a marker to predict the invasiveness and metastasis of PBC.

## Introduction

Primary breast cancer (PBC) is one of the most common malignancies amongst females, accounting for 23% of total cancer diagnoses and 14% of all cancer-associated mortalities in females worldwide (1). Significant progress has been made in the treatment of primary tumors, and multiple randomized trials have demonstrated the efficacy of adjuvant chemotherapy and hormonal treatment in prolonging the survival of patients with breast cancer (2–4). Current treatment strategies include wide local excision and radiotherapy or mastectomy, depending on the size of the tumor. In addition, the majority of patients receive postoperative radiotherapy (5). As for adjuvant systemic therapy, endocrine-responsive tumors are treated with tamoxifen or aromatase inhibitors with adjuvant chemotherapy, while tumors that are endocrine non-responsive are treated with chemotherapy (6). However, despite significant progression in improving early detection and treatment strategies, 30–50% of patients are at high risk of metastasis and 10–15% of patients develop distant metastases within 10 years of initial diagnosis (7). The most significant predictors of PBC disease recurrence and outcome, include tumor size, histological grade, lymph node involvement, expression of estrogen and/or progesterone receptors, human epidermal growth factor receptor 2 expression and the presence of circulating tumor cells (8,9).

Metastasis involves local tissue invasion by tumor cells via cytoskeletal reorganization, migration of cells through the tissue into the vascular or lymphatic system via lamellipodia and establishment of secondary tumors at distant sites via the activity of adhesion proteins (10). The management of metastasis currently remains a major challenge for patients with PBC and there has been a recent focus on targeting signaling pathways between the primary tumor and disseminated metastases (11).

Rac, a member of the Rho family of GTPases, has been shown to mediate multiple signaling pathways involved in organization of the actin cytoskeleton, as well as invasion and migration of tumor cells via p21-activated kinases (PAKs) (12–14). PAKs have been reported to phosphorylate and activate LIM kinase, which subsequently activates cofilin in order to regulate the turnover of actin filaments (15).

Nischarin, a novel tumor suppressor, was initially identified as an ~190 kDa cytosolic protein, which mapped to 3p21 (16,17). Nischarin was found to bind to the α5 subunit of integrins, and inhibited Rac-mediated cell motility and invasion in breast and colon epithelial cells (18–21). Notably, IRAS, the human homolog of Nischarin, was described as an imidazoline receptor (22) with anti-apoptotic activity (23,24). Nischarin mRNA expression levels have been reported to be significantly higher in the brain and kidney compared with those in the heart, liver, lung and skeletal muscle (19). Additionally, Nischarin expression was recently revealed to be widely distributed in rat brain tissue, particularly in the cerebral cortex and hippocampus, and is hypothesized to exhibit a significant role in neuronal migration (25).

The expression levels of Nischarin were previously demonstrated to be significantly higher in normal breast tissue compared with breast cancer tissue, and the loss of Nischarin expression in breast cancer tissue is hypothesized to be due to a loss of heterozygosity (26). However, to the best of our knowledge, the role of Nischarin in breast cancer metastasis has previously only been studied *in vitro* (27) and the mechanisms underlying Nischarin-mediated inhibition of metastasis remain to be elucidated. In the present study, the expression of Nischarin protein in PBC and adjacent normal tissues was evaluated. The correlation between Nischarin expression levels and breast cancer metastasis was also examined, in order to aid the elucidation of the role of Nischarin in the occurrence, development and metastasis of PBC.

## Materials and methods

### Reagents

The NISCH ELISA kit was purchased from USCN Life Sciences (Wuhan, China). CHAPS, Tris buffer and urea were obtained from Bio-Rad Laboratories, Inc. (Hercules, CA, USA) and the bicinchoninic acid (BCA) protein quantification kit was purchased from Shanghai Sangong Biotech Co., Ltd. (Shanghai, China).

### Sample collection

A total of 60 primary cancer tissues and the corresponding adjacent normal tissues were collected from patients with breast cancer during modified radical mastectomy at the Department of Breast Surgery of the Zhejiang Cancer Hospital (Hangzhou, China) between February 2008 and February 2010. Tissues were stored at −70°C prior to use. Seven tissue samples were classified as grade I ductal carcinoma, 33 tissue samples were grade II and 20 tissue samples were grade III. Pathological examination indicated the presence of lymph node metastasis in 30 of the tissue samples, while 30 tissue samples were negative for lymph node metastasis. Of the 30 samples with lymph node metastasis, 21 tissue samples had <3 lymph node metastases, while nine tissue samples had >3 lymph node metastases. All 60 tissue samples were identified as invasive ductal carcinoma in post-operative pathological examinations. Cancer stages were graded according to the AJCC Cancer Staging Manual (28,29) and histological grade was determined according to the Nottingham Combined Histological Grade (30).

None of the patients had received chemotherapy or physical therapy prior to surgery. The study was reviewed and approved by the Ethics Committee of Zhejiang Cancer Hospital (Huangzhou, China). Written informed consent was obtained from all patients involved in the study.

### Protein extraction

Tissue samples were washed three times in normal saline and residual water was removed with a filter. Tissue samples were resuspended in 200 *μ*l lysis buffer (4% CHAPS, 30 mM Tris buffer and 8 M urea; pH 8.5) and sonicated (JY92-II DN; Ningbo Scientz Biotechnology Co., Ltd, Zhejiang, China) on ice at 200 W for a total of 150 sec, with an interval of 10 sec between 10 sec bursts. The sonicated samples were centrifuged at 12,000 × g for 30 min at 4°C and the protein concentration of the supernatant was determined.

### ELISA

Nischarin expression levels were determined using a NISCH ELISA kit (containing detection solution B, substrate solution and stop solution; USCN Life Science, Inc., Wuhan, China) according to the manufacturer’s instructions. Briefly, 100 *μ*l standards (5, 2.5, 1.25, 0.625, 0.312 and 0.156 ng/ml) or samples were incubated at 37°C for 2 h. The wells were washed three times with 350 *μ*l washing solution and were subsequently incubated with 100 *μ*l of freshly prepared detection solution B for 30 min at 37°C. The wells were washed five times and then incubated for 15–25 min in the dark with 90 *μ*l substrate solution at 37°C. The reaction was stopped by the addition of 50 *μ*l stop solution and the absorbance (OD) was measured at 450 nm using a SpectraMAX M3 microplate reader (Molecular Devices, Sunnyvale, CA, USA). Standard curves were constructed and the following regression equation was calculated in order to determine the concentration of the samples: Concentration=5(OD)-0.03, R^2^=1.

### Statistical analysis

Continuous variables are presented as the mean ± standard deviation. Differences in mean age and Nischarin concentrations between patients with and without lymph node metastasis were analyzed by independent two-sample t-tests. Differences in mean concentrations of Nischarin between cancer tissues and adjacent noncancerous tissues were analyzed by paired t-tests. Differences in the mean concentrations of Nischarin in cancer tissues from various grades were analyzed by nonparametric Kruskal Wallis test, due to the low number of grade I and III cases. All statistical assessments were two-sided and P<0.05 was considered to indicate a statistically significant difference. Statistical analyses were performed with SPSS 18.0 statistics software (SPSS Inc., Chicago, IL, USA).

## Results

### Clinical characteristics

The average age of the 60 patients was 51.1±9.9 years. There was no significant difference in age between patients with and without lymph node metastasis (50.9±9.6 vs. 51.3±10.4 years; P=0.898). All clinical characteristics of the patients evaluated, including cancer stage and histological grade, are summarized in Table I.

### Nischarin concentration is lower in breast cancer tissues

**Figure 1:**
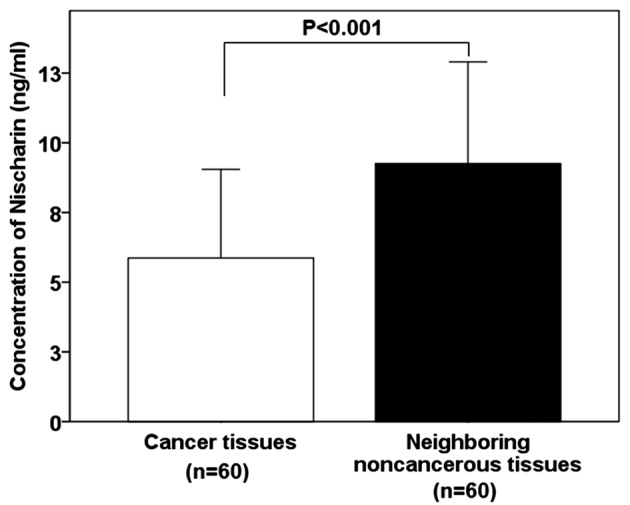
Comparison between Nischarin protein expression levels in primary breast cancer tissues and adjacent noncancerous tissues.

The mean protein concentration of Nischarin was demonstrated to be significantly lower in breast cancer tissues compared with that of the adjacent non-cancerous tissues (5.86±3.19 vs. 9.25±3.65 ng/ml; P<0.001; Fig. 1).

### Nischarin concentration is lower in patients with lymph node metastasis

**Figure 2:**
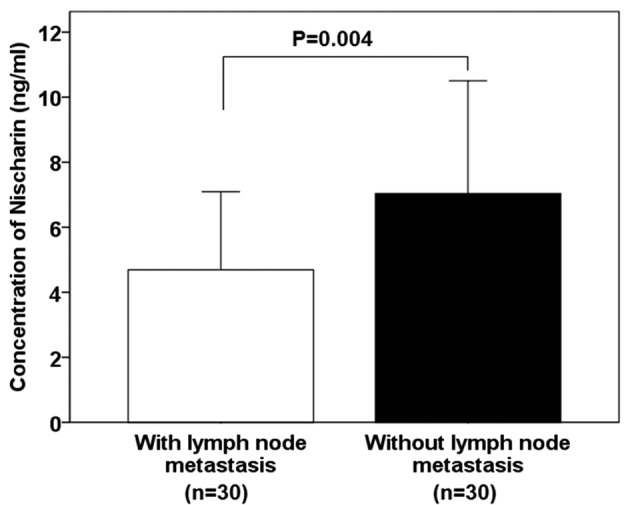
Comparison of Nischarin protein expression levels in primary breast cancer patients with and without lymph node metastasis.

The mean concentration of Nischarin protein was found to be significantly lower in tissues from patients with lymph node metastasis compared with those of patients without lymph node metastasis (4.69±2.40 vs. 7.04±3.47 ng/ml; P=0.004; Fig. 2).

### Nischarin concentration does not differ between PBC grades

**Figure 3:**
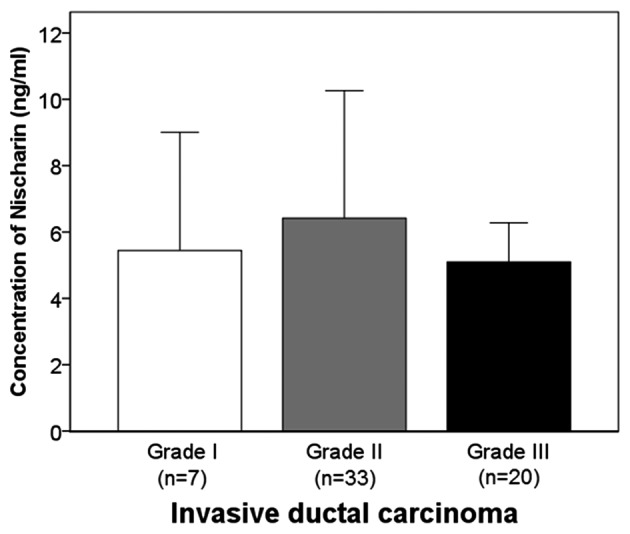
Comparison of Nischarin protein expression levels in primary breast cancer tissues from invasive ductal carcinoma of different grades.

The expression of Nischarin protein in cancer tissues from various grades of invasive ductal carcinoma were evaluated, and no significant differences were detected between each grade (grade I, 5.44±3.57; grade II, 6.42±3.85; grade III, 5.10±1.18 ng/ml; P=0.765; Fig. 3).

## Discussion

In the present study, the expression levels of Nischarin in breast cancer tissues were compared with those in adjacent noncancerous tissues. Nischarin expression was also compared between patients with and without lymph node metastasis, and in patients with varying grades of breast cancer. The results indicated that: i) Nischarin expression was significantly lower in breast cancer tissues compared with that of normal tissues; ii) Nischarin expression levels were significantly lower in patients with lymph node metastasis compared with those of patients without lymph node metastasis; and iii) there was no significant difference in Nischarin expression levels between patients with grades I, II or III breast cancer.

Integrins exhibit a critical role in multiple signal transduction processes in order to regulate the cell cycle and cell death (31,32). Upregulation of integrin α5β1 expression was demonstrated to inhibit tumor cell growth (33) and protect cells against mitogen deprivation-induced apoptosis (34). Nischarin has been suggested to be involved in the inhibition of tumor cell growth via upregulation of the expression of the α5 subunit, reducing the phosphorylation of focal adhesion protein tyrosine kinase and decreasing the Rac GTP load (26). Low Nischarin expression levels may therefore lead to increased tumor cell proliferation and reduced cell apoptosis, resulting in carcinogenesis.

The interaction of Nischarin with the α5 subunit of integrins to regulate cell migration suggested that Nischarin may have a role in mediating the metastasis of malignancies (26,27). Concurrently, the overexpression of Nischarin was shown to result in inhibition of cell migration of fibroblasts *in vitro*, although this inhibition was not associated with cytotoxicity (19). In addition, short interfering RNA-mediated silencing of Nischarin expression was observed to stimulate fibroblast migration (21). Overexpression of Nischarin in MCF-7 breast cancer cells also resulted in the inhibition of cell migration, as indicated by a Transwell assay, although Nischarin overexpression did not significantly influence cell adhesion (35). Studies observing the mechanisms underlying Nischarin-mediated inhibition of cell migration demonstrated significantly higher rates of migration in Rac-overexpressing cells compared with those of control cells, and this migration was abrogated by the simultaneous overexpression of Nischarin. Nischarin was also shown to directly interact with Rac and PAK1, suggesting that Nischarin inhibited migration by selectively interfering with the Rac-mediated signaling pathways, which regulate cell migration via PAK (20,36). Notably, Nischarin selectively inhibited migration of MCF-7 cells induced by PAK, but not migration induced by MEK kinase 1, a Rac effector in the c-Jun N-terminal kinase pathway, or migration induced by MEK1, which is an effector in the Ras-Raf-MEK-extracellular-signal-regulated kinase pathway (20). A study also indicated that Nischarin was able to regulate Rac1 signaling pathways independent of PAK1 (37).

Further studies aiming to elucidate the mechanisms underlying Nischarin-mediated regulation of cell migration and invasion identified a direct association between Nischarin and LIM kinase (LIMK), which is a downstream effector of PAK and is known to have a significant role in cell motility, cell invasion and the G_2_/M checkpoint of the cell cycle (38–40). LIMK has been reported to regulate the phosphorylation and dephosphorylation of cofilin, which is an important determinant of actin-based cell motility (41,42). Direct binding of Nischarin with LIMK has been shown to inhibit LIMK activity, cofilin phosphorylation and LIMK-mediated invasion of MCF-7 breast cancer cells (43). Nischarin has also recently been shown to directly associate with tumor suppressor LKB1 in breast cancer cells. The suppression of Nischarin and LKB1 in these cells resulted in increased phosphorylation of PAK1 and LIMK1, and upregulation of Cyclin D1 and CDK4 expression, resulting in enhanced cell migration and tumor growth (27).

MicroRNAs (miRs) are small noncoding endogenous RNAs that negatively regulate gene expression at the transcriptional or translational level by binding to the 3′-untranslated region of their target mRNAs (44). The expression of miR23b and miR27b, which are highly expressed in breast cancer cells, was shown to be inversely correlated with Nischarin expression levels. Furthermore, Nischarin was shown to negatively regulate the expression of miR23b/27b via the inhibition of NFκB phosphorylation (45). Further investigation into the Nischarin signaling pathways is required in order to elucidate the mechanisms underlying Nischarin-mediated inhibition of tumor cell migration and metastasis in PBC and other types of cancer.

Nischarin has also been demonstrated to be significantly downregulated in human breast cancer tissues compared with normal tissues in patients with breast cancer from the USA, and the overexpression of Nischarin in MDA-MB-231 breast cancer cells significantly inhibited metastasis, suggesting that Nischarin may function as a tumor suppressor (27). Nischarin expression was also associated with more advanced tumor grades and a decrease in survival (27). In the present study ELISA analysis revealed significantly lower expression levels of Nischarin in breast cancer tissues compared with adjacent normal tissues in patients with PBC. Additionally, Nischarin expression was found to be significantly lower in patients with lymph node metastasis compared with that of patients without lymph node metastasis, suggesting that Nischarin expression levels may be a reliable indicator for the prediction of the invasiveness and metastatic potential of breast cancer. In the present study, no significant correlation was observed between tumor grade and Nischarin expression levels. The results indicated the reproducibility, high sensitivity, specificity and ease of use of the Nischarin ELISA assay, suggesting that it may be efficiently used in clinical practice.

**Figure 4:**
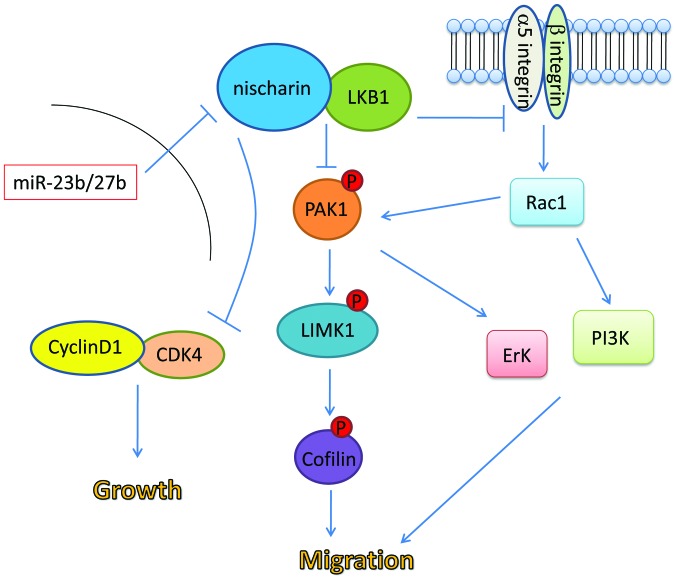
Schematic diagram suggesting the role of Nischarin in cell migration and tumor growth. LKB1, liver kinase B1; PAK1, p21-activated kinase 1; LIMK1, LIM domain kinase 1; CDK4, cyclin-dependent kinase 4; Erk, extracellular-signal-regulated kinase; PI3K, phosphoinositide 3-kinase; miR, microRNA; p, phosphorylated.

Based on the data available from previous studies as well as the results of the present study, a potential model of the role of Nischarin in cell migration and tumor growth was suggested (Fig. 4). Binding of Nischarin to LKB1 may inhibit integrin-mediated activation of the Rac1 pathway, which promotes cell migration. The Nischarin-LKB1 interaction may also inhibit phosphorylation and activation of the PAK1-LIMK1-cofilin pathway, which promotes cell migration. Finally, it is possible that the Nischarin-LKB1 interaction may inhibit cell cycle progression via inhibition of the Cyclin D1/CDK4 complex. High expression levels of miR23b/27b in breast cancer cells may inhibit the interaction between Nischarin and LKB1, abrogating the tumor suppressor effects of Nischarin.

In conclusion, the results of the present study revealed that Nischarin expression was significantly lower in breast cancer tissues compared with adjacent normal tissues in Chinese patients with PBC. To the best of our knowledge, the present study was the first to demonstrate that Nischarin expression levels were significantly lower in patients with lymph node metastasis compared with patients with no lymph node metastasis. A major limitation of the present study was that the mechanisms underlying the role of Nischarin in the inhibition of metastasis were not investigated. It may also be important to investigate the role of Nischarin in different types of cancer. Further studies are required to verify the role of Nischarin as a prognostic marker for breast cancer metastasis.

## Figures and Tables

**Table I:** Clinical characteristics of patients with primary breast cancer.

Characteristic	LN metastasis (n=30)	No LN metastasis (n=30)	Total (n=60)
Age (years)^a^	50.9±9.6	51.3±10.4	51.1±9.9
Gender, female^b^	30 (100)	30 (100)	60 (100)
Cancer stage^b^			
Ia	0 (0)	3 (10.0)	3 (5.0)
Ib	0 (0)	0 (0.0)	0 (0)
IIa	1 (3.3)	26 (86.7)	27 (45.0)
IIb	18 (60.0)	1 (3.3)	19 (31.7)
IIIa	6 (20.0)	0 (0)	6 (10.0)
IIIb	1 (3.3)	0 (0)	1 (1.7)
IIIc	4 (13.3)	0 (0)	4 (6.7)
Histological grade^b^			
I	2 (6.7)	5 (16.7)	7 (11.7)
II	21 (70.0)	12 (40.0)	33 (55.0)
III	7 (23.3)	13 (43.3)	20 (33.3)

Values are expressed as

a mean ± standard deviation;

b n (%). LN, lymph node.
